# Early Changes in Androgen Levels in Individuals with Spinal Cord Injury: A Longitudinal SwiSCI Study

**DOI:** 10.3390/jcm11216559

**Published:** 2022-11-04

**Authors:** Oche Adam Itodo, Peter Francis Raguindin, Jens Wöllner, Inge Eriks-Hoogland, Xavier Jordan, Margret Hund-Georgiadis, Taulant Muka, Jürgen Pannek, Jivko Stoyanov, Marija Glisic

**Affiliations:** 1 Swiss Paraplegic Research, 6207 Nottwil, Switzerland; 2 Institute of Social and Preventive Medicine, University of Bern, 3012 Bern, Switzerland; 3 Graduate School for Health Sciences, University of Bern, 3012 Bern, Switzerland; 4 Swiss Paraplegic Centre, 6207 Nottwil, Switzerland; 5 Clinique Romande de Réadaptation, 1950 Sion, Switzerland; 6 Center for Neurorehabilitation and Paraplegiology, REHAB Basel, 4055 Basel, Switzerland; 7 Epistudia, 3011 Bern, Switzerland

**Keywords:** spinal cord injury, rehabilitation, androgens, testosterone, sex hormone-binding globulin, dehydroepiandrosterone, dehydroepiandrosterone sulfate

## Abstract

We aimed to explore longitudinal changes in androgen levels in individuals with spinal cord injury (SCI) within initial inpatient rehabilitation stay and identify clinical/injury characteristics associated with hormone levels. Linear regression analysis was applied to explore the association between personal/injury characteristics and androgen hormones (total testosterone, free testosterone, sex hormone-binding globulin (SHBG), dehydroepiandrosterone (DHEA), and dehydroepiandrosterone sulfate (DHEA-S)) at admission to rehabilitation. Longitudinal changes in androgen levels were studied using linear mixed models. Analyses were stratified by sex and by injury type. We included 70 men and 16 women with SCI. We observed a non-linear association between age, time since injury, and androgens at baseline. At admission to initial rehabilitation, mature serum SHBG (full-length, protein form which lacks the N-terminal signaling peptide) was higher, while DHEA and DHEA-S were lower among opioid users vs. non-users. Serum levels of total testosterone and DHEA-S increased over rehabilitation period [β 3.96 (95%CI 1.37, 6.56), *p* = 0.003] and [β 1.77 (95%CI 0.73, 2.81), *p* = 0.01], respectively. We observed no significant changes in other androgens. Restricting our analysis to men with traumatic injury did not materially change our findings. During first inpatient rehabilitation over a median follow up of 5.6 months, we observed an increase in total testosterone and DHEA-S in men with SCI. Future studies need to explore whether these hormonal changes influence neurological and functional recovery as well as metabolic parameters during initial rehabilitation stay.

## 1. Introduction

A decline in androgen hormones and abnormalities of the hypothalamic-pituitary-gonadal (HPG) axis has been repeatedly reported in individuals with chronic spinal cord injury (SCI), with more than 40% of men having testosterone levels below normal age-specific cut-offs [1,2]. Deficiency of androgen hormones primarily influences sexual function and fertility [3]. Low testosterone, in addition, may accelerate the aging processes in individuals with SCI by promoting development of sarcopenic obesity, metabolic disorders and hyperinflammatory state [4,5]. Dehydroepiandrosterone (endogenous steroid hormone precursor), and its sulphated ester (DHEA-S) have been linked with immune function and inflammation, bone metabolism and physical strength in frailty, as well as risk of diabetes [6,7,8]. Sex hormone-binding globulin (SHBG), which primary role is to bind testosterone, was inversely associated with insulin resistance, inflammation, diabetes, and metabolic syndrome [9,10,11,12,13]. In addition, in animal models of SCI, endogenous and exogenous estradiol were linked with improved recovery post-injury in both sexes [14,15], while androgens (testosterone and DHEA), on the other hand, may excrete sex-specific effects in SCI (e.g., high testosterone in women, and low testosterone in men have mirroring effects on metabolism) [15,16].

Although testosterone decline occurs earlier in life in men with SCI as compared to able-bodied individuals (ABI) [17,18], the evidence on association between injury duration and testosterone levels is contradicting [18,19]. Comprehensive studies in the subacute phase of the injury are scarce, thus making it difficult to understand the trajectory of early changes in androgen levels following the injury, which is of utmost importance for understanding the role of androgens in modifying metabolic changes, rehabilitation outcomes and functioning post-injury [20,21]. In addition, current studies were predominantly focused on testosterone and HPG axis, while studies on DHEA, and DHEA-S, which is the most abundant steroid hormone with important biological functions, remain scarce. Further, despite important physiological role of androgens in females, studies in women are uncommon [22,23]. Women were often purposely excluded from analyses, further widening the literature gap [24]. Finally, previous studies often did not account for SCI characteristics, body morphology, physical activity, underlying comorbidities and medication in their analysis, which all have been shown to influence hormone levels in SCI [1,20,25,26].

Thus, in the current study, we aimed to: (i) explore the longitudinal changes in androgen levels in men and women with SCI during initial rehabilitation stay and (ii) identify clinical characteristics associated with hormone levels by using a multicenter SCI cohort in Switzerland.

## 2. Materials and Methods

### 2.1. Study Setting

The inception cohort of the Swiss Spinal Cord Injury cohort (SwiSCI) is a prospective multicenter study that recruits study participants across four major rehabilitation centers in Switzerland, namely, Swiss Paraplegic Center (Nottwil), Clinique Romande de Readaptation (Sion), Balgrist Spine Center (Zurich), and Basel Rehabilitation Clinic (Basel) [27]. Study participants were involved in interdisciplinary rehabilitation approach tailored to person’s specific needs and aimed to optimize one’s functioning. The SwiSCI inception cohort collects numerous demographic, biopsychosocial and clinical parameters at five time points after the date of SCI diagnosis: at 28 days (range 16–40 days, T1), 84 days (70–98 days, T2), 168 days (150–186 days, T3), at discharge (10–0 days before discharge, T4) and one year after diagnosis. In addition, the SwiSCI biobank provides a platform for conducting research within the Inception Cohort of SwiSCI by cryopreserving serum, plasma, and peripheral blood mononuclear cells (PBMC), RNA, DNA and urine for research purposes. Biobank sampling (at T1 and T4), started on the 27 of June 2016 in the largest center (Nottwil), followed by 2 other centers (Basel and Sion) on the 23 of August 2018 and the 15 of January 2019, respectively. Detailed information on the study design and collected data can be found elsewhere [27]. 

### 2.2. Study Population, Inclusion and Exclusion Criteria

The SwiSCI enrolled individuals aged over 16 years with traumatic or non-traumatic SCI receiving their first specialized rehabilitation in Switzerland. Individuals with injuries attributable to either a congenital condition, neurodegenerative disorder, or Guillain-Barré syndrome or who had a new SCI in the context of palliative care were excluded from the study. All SwiSCI study participants who provided serum samples at both the beginning and end of rehabilitation between 27 of June 2016 and 20 of January 2021, were eligible for inclusion. In addition to SwiSCI exclusion criteria, we excluded individuals with congestive heart failure, and inflammatory bowel disease and sex hormone therapy users. 

### 2.3. Study Measures

#### Androgen Hormones

The SwiSCI study participants had blood drawn between 7:00 a.m and 2:00 p.m from the antecubital vein for serum processing which was spun at 1800× *g*, separated, and stored at −80 °C until batch processing for subsequent quantification of androgens (that were not routinely measured in the SwiSCI study). All hormones were measured using Enzyme-Linked Immunosorbent Assay (ELISA). Total testosterone, free testosterone, and SHBG were measured using ELISA kits (Abcam, Lucerna-Chem AG, Luzern, Switzerland, cat.no:ab174569, cat.no:ab178663 and cat.no:ab260070, respectively). DHEA and DHEA-S were measured using ELISA kits (Abnova, Lucerna-Chem AG, Luzern, Switzerland, cat.no:KA0315 and cat.no:KA0920, respectively). All plates were scanned on a Beckman Coulter, Inc., Brea, CA, USA, multimode analysis software and the final levels of the various sex hormones were determined on Myassays Ltd. online database [28] using a four parameter logistic fit according to manufacturer’s instructions. Details are provided in Supplementary Table S1. 

### 2.4. Clinical and SCI Characteristics

Demographic characteristics such as age at baseline, sex, information on comorbidities and medication use, duration of injury and SCI characteristics were derived from the patient’s medical records. The level of injury was classified as tetraplegia (at level C2–C8) and paraplegia (level T1–S5), and the completeness of injury into complete motor injury (AIS A and B) and incomplete (AIS C and D) based on the International Standards for Neurological Classification of Spinal Cord Injury (ISNCSCI) [29]. “International SCI Basic Data Sets” suggested by the International Spinal Cord Injury Society was used to collect information on sexual dysfunction [30]. Waist circumference (WC) was measured using a pliable tape measure expressed in cm. Body mass index (BMI) was computed employing the standard formula [weight in kilograms/(height in meters)^2^]. 

### 2.5. Power Calculation

Three sources helped guide the estimation of the sample size for the study: (1) previous similar research, (2) general statistical principles, and (3) the “*Gpower*”(ver. 3.1.9.7; Heinrich-Heine-Universität Düsseldorf, Düsseldorf, Germany; http://www.gpower.hhu.de/ accessed on 4 October 2022) power analysis program Sample sizes in similar research ranged from 20 to 92 SCI participants [20,21,31]. General consensus on principles of statistics suggests multiple regression analyses should have 10 participants per independent variable (6 variables in our case). The program “*Gpower*” estimated a minimum of 57 subjects was needed to achieve 80% power with an anticipated large Cohen’s f-square effect size of 0.35 for multiple regression.

### 2.6. Statistical Analyses

We summarized our data using median and interquartile range (IQR), or counts with percentages, as appropriate. Wilcoxon signed-rank test (continuous data) or Chi-squared test (nominal/dichotomous data) were used to determine differences in demographic and clinical profiles between men and women. At baseline, we used linear regression analysis to determine the association between independent variables: injury characteristics (etiology, level and completeness), opioid use and corticosteroid use and androgens (dependent variables). We fit restricted cubic splines to describe the trend between age and androgen hormones, and between time since injury and androgens. We applied both the paired t-test and multilevel mixed model using random intercept and residual maximum likelihood estimation to determine the longitudinal changes in androgens across the period of inpatient rehabilitation. The model was adjusted for age, BMI, level of injury, completeness of injury, time since injury, opioids use, and corticosteroids use in [32]. All analyses were stratified according to sex and were performed using Stata 16.1 (StataCorp LLC, College Station, TX, USA) for Windows. All computations were done using two-tailed tests, and a *p*-value of <0.05 was considered statistically significant. 

## 3. Results

### 3.1. Baseline Characteristics

**Figure 1:**
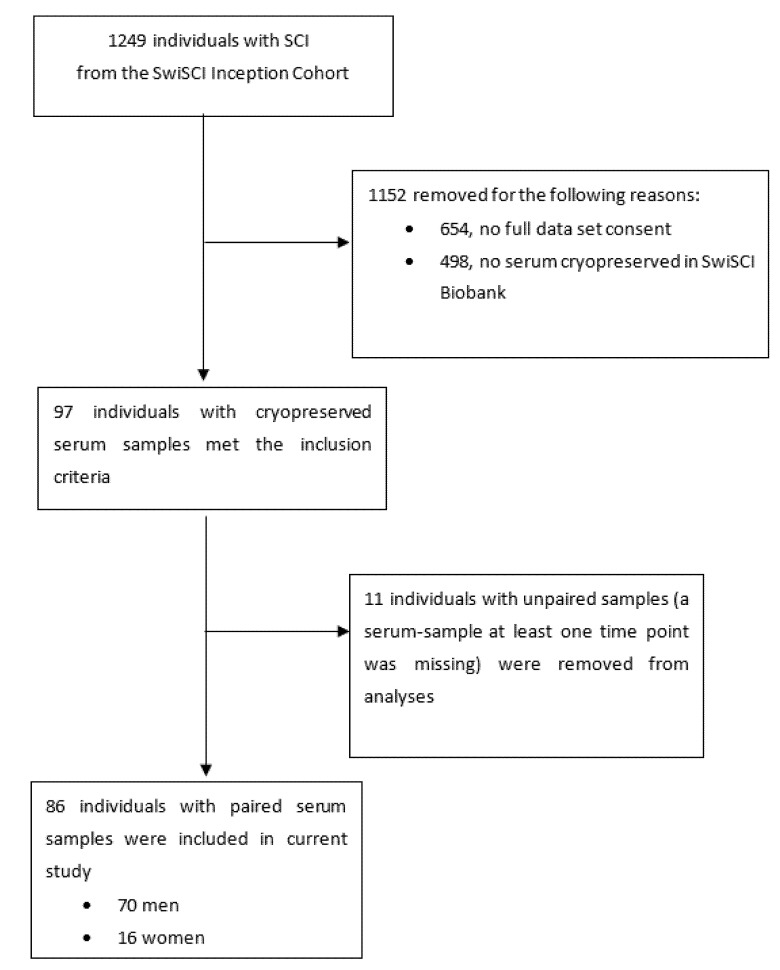
Flowchart of study participants.

A total of 86 SCI individuals, 70 men (81%) and 16 women (19%), were included in the study (Figure 1) and the median age of the study population was 51 years (IQR 36–64).

The majority of study participants (n = 61, 70.9%) had traumatic SCI, 33 (38.4%) individuals had a motor complete injury and the median duration of injury at the moment of admission to first inpatient rehabilitation was 15.5 days (IQR 10–27). In Table 1, we present the most important clinical characteristics of study participants at admission to rehabilitation stratified by sex. Besides statistically significant differences in causes of TSCI and body weight, other personal and clinical characteristics did not differ between sexes. In men, median total and free testosterone levels were 12.5 nmol/L (IQR 7.9–17.7) and 27.5 pmol/L (IQR 16.9–36.6), respectively. Total and free testosterone levels were significantly lower in women (1.9 nmol/L (IQR 1.4–2.5) and 2.9 pmol/L (2.3–3.7)). No differences between sexes were observed in other androgens. Median DHEA level was 20.2 nmol/L (IQR 12.8–40.2) in men and 18.9 nmol/L (IQR 15.7–35.0) in women, while median DHEA-S was 3.6 µmol/L (IQR 1.6–6.9) and 3.1 µmol/L (IQR 1.5–4.6) in men and women, respectively. SHBG levels were 2558 pg/mL (IQR 2053–2777) and 2890 pg/mL (IQR 2020.5–3947) in men and women, respectively. We provide details of the medical conditions associated with NTSCI in Table S2.

Forty-eight men (88.89%) expressed willingness to respond to questions related to sexual function. Orgasmic function, psychogenic erection, reflex erection, and ejaculation were either absent or reduced among the majority of men who responded to the questionnaire, Supplementary Figure S1.

### 3.2. Association between Clinical Characteristics and Androgens at Baseline

The association between age and time since injury and androgen hormones was non-linear in both men and women, and in men with motor complete traumatic SCI, these associations were described using restricted cubic splines. Results can be found in the online supplement (Supplementary Figures S2–S6). In regression analysis, DHEA-S levels were lower among individuals with incomplete as compared to complete injury (β −1.78 (95%CI −3.43, −0.12) *p* < 0.05). Men who used opioids as compared to men who did not receive opioids had lower DHEA (β −9.02 (95%CI −17.01, −1.03) *p* < 0.001), DHEA-S (β −2.18 (95%CI −3.58, −0.78) *p* < 0.001) and higher SHBG levels [β 557.76 (95%CI 281.48, 834.03) *p* < 0.001). No differences were observed among individuals with traumatic and non-traumatic injury, tetra- and paraplegia, users, and non-users of corticosteroids (Table 2). When restricting our analysis to men with traumatic SCI, results did not materially change (Supplementary Table S3). Women with non-traumatic as compared to traumatic injury had lower DHEA (β −18.5 (95%CI −35.51, −0.58) *p* < 0.05). SHBG was higher among opioid users (β 1158.41 (95%CI 310.59, 2006.23) *p* < 0.05), whereas lower trends of DHEA and DHEA-S seen among opioid users did not reach statistical significance, Table 2.

### 3.3. Longitudinal Changes in Androgen Levels

**Figure 2:**
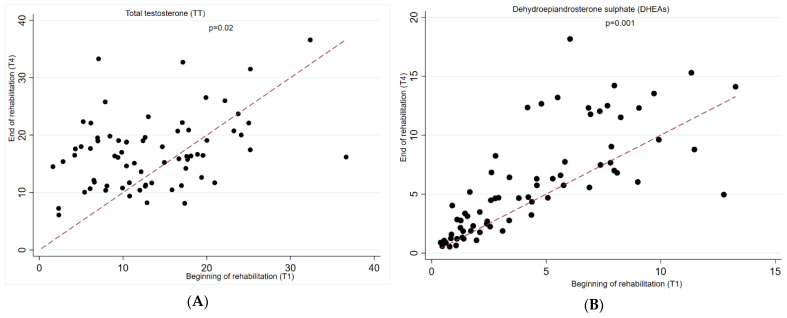
Bivariate plots of changes in androgen sex hormone levels in men with spinal cord injury. The bivariate plot shows individual changes in (**A**) total testosterone and (**B**) DHEA-S comparing the beginning and end of the rehabilitation period. A dotted line represents a line of no change. Every circle represents a person. A circle above the line indicates that a person had a higher level of androgen at follow-up as compared to baseline, whereas a circle below the line indicates that a person had a lower level of androgen at follow-up as compared to baseline.

In the fully adjusted linear mixed model (age, BMI, level and completeness, time since injury, use of opioids and corticosteroids), we observed a significant increase in total testosterone (β 3.96 (95%CI 1.37, 6.56) *p* = 0.003) and DHEA-S (β 1.77 (95%CI 0.73, 2.81) *p* = 0.001) in men with SCI when comparing beginning and end of rehabilitation period. We observed no significant changes in free testosterone, DHEA, or SHBG in men with SCI, Table 3. The results remained stable when restricting our analyses to men with traumatic injury, as well as when comparing the results of linear mixed models and paired t-test and Wilcoxon signed rank test (Supplementary Tables S4 and S5). In women, we observed a significant decrease in mean DHEA levels comparing the beginning and end of the rehabilitation period (β −11.91 (95%CI −24.39, −4.11) *p* < 0.001). No significant longitudinal changes were observed among other hormones, Table 3. Figure 2, and Supplementary Figures S7 and S8 show individual trajectories in changes in androgen levels.

## 4. Discussion

Over a period of initial rehabilitation stay (between admission or within 16–40 post-injury to up to 10 days before discharge), total testosterone and DHEA-S levels increased significantly, while we observed no significant changes in other hormones (free testosterone, SHBG, or DHEA). At admission to initial rehabilitation, serum SHBG was higher, while DHEA and DHEA-S were lower among opioid users vs. non-users. We observed a non-linear association between age and injury duration and hormone levels. Our findings on women should be interpreted with caution, considering that only 16 women were available for analysis. 

### 4.1. Androgen Changes during Initial Inpatient Rehabilitation

A recent meta-analysis including 37 observational studies reported significantly lower total testosterone among individuals with chronic SCI compared to ABI, with no differences in free testosterone nor SHBG between the two groups [33]. Low testosterone levels reported in the literature varied from 10% to more than 70% of men with SCI [34]. Higher prevalence of testosterone deficiency was reported among individuals with motor complete (as compared to motor incomplete) and cervical (as compared to thoracic/lumbosacral) injuries and among individuals using narcotic medications for pain management [17,19,35]. Although scarce, studies in the subacute phase of the injury show similar patterns in early testosterone changes. Naftchi et al. measured sex hormones in urine once a week for four months starting from the onset of the injury [21]. Individuals with paraplegia (as compared to age-matched ABI) had lower luteinizing hormone (LH) and follicle-stimulating hormone (FSH) for two weeks and lower levels of testosterone for six weeks after the injury, subsequently reaching normal levels. In individuals with tetraplegia, serum testosterone concentrations remained significantly lower than those of the controls during the entire 4-month testing period [21]. In a study by Schopp et al., the time since injury was associated with testosterone levels, with those having an acute injury being more likely to have low testosterone than those with a chronic injury [20]. Similarly, among men recruited at the inpatient rehabilitation unit, mean total and free testosterone levels were lower among individuals ≤12 months post-injury as compared to individuals >12 post-injury [31]. Due to variability in methods used to measure androgens (ELISA in current study, Levy and Schwartz (1973) modification of the Bradlow (1968) method or chemiluminescent microparticle immunoassay and radioimmunoassay in previous studies) it remains challenging to compare the prevalence of low androgen levels across studies. European Academy of Andrology (EAA) guidelines on hypogonadism in males suggest liquid chromatography-mass spectrometry (LC-MS) as a preferred method for androgen assessment [36]. They further recommend using standardized methods, such as immunoassay, for research purposes, as they show high correlation with LC-MS/MS within the adult male testosterone range (although they offer less precision in the hypogonadal range). In our study, at admission to rehabilitation low total (<8 nmol/L or <231 ng/dL) and free testosterone (<220 pmol/L or <6.3 ng/dL) were seen among 27% and 36% of men with SCI.

We observed significant increases in total testosterone and DHEA-S over rehabilitation, while free testosterone, SHBG and DHEA were not significantly changed. Lack of significant variations in free testosterone could be explained by low albumin levels observed in the acute phase of the injury. Testosterone is predominantly bound to SHBG (60–70% of total testosterone), while around 30% to 40% is loosely bound to albumin [37]. Therefore, lower albumin leaves a higher amount of free testosterone in circulation. Meaning that at baseline, free testosterone levels in blood may be overestimated. Further, our findings may be at least partially explained by the decreased prevalence of opioid medication use over the period of rehabilitation (which decreased from 32.9% to 11.4%). Indeed, at baseline, we observed significantly lower levels of DHEA and DHEA-S and higher levels of SHBG among opioid users as compared to non-users (no differences observed in free and total testosterone). In addition, elevated levels of corticosterone/cortisol (either exogenous or endogenous) may drive a decrease in testosterone acutely following injury [38]. Cortisol levels typically reverse to normal within 6 months of injury which is in line with the improvement in hormonal status within a year since injury as reported earlier or within on average 5.6 months of follow-up in our study [39]. Finally, an increase in testosterone levels observed in our study may be as well driven by a thorough exercise prescription within a rehabilitation program which may stimulate production of testosterone [40].

### 4.2. Clinical Implications of Our Findings and Directions for Future Research

Within weeks since injury, testosterone level decreases, and thereafter may reach normal values within 4–6 months post-injury, which is fairly supported by our study that reported a significant increase in total testosterone and DHEA-S prior to discharge from first inpatient rehabilitation. The evidence in chronic SCI indicates that men with SCI may be a target population for testosterone deficiency screening. However, the timing of such screening strategies remains debated. For example, Sullivan et al. suggested implementing a routine testosterone deficiency screening strategy among men with SCI beginning at least 1-year post-injury and/or by the 3rd decade of life [34]. To develop the SCI-specific testosterone deficiency screening guidelines, a comprehensive systematic overview of the literature engaging the GRADE approach [41] should identify subgroups of SCI individuals that are at a higher risk for developing testosterone/androgen deficiency and provide a rationale for early testosterone screening. In addition, sex steroids, especially 17β-estradiol and progesterone, showed neuroprotective effects and led to improvement of functional deficits in animal models of SCI [14]. Future human studies should investigate whether sex steroids may influence changes in the metabolic milieu in subacute injury and influence functional recovery during rehabilitation. In line with this, testosterone treatment alone or combined with resistance training may be a reasonable strategy to slow down the bone loss and fat accumulation following the injury and improve muscle size, strength and contractile properties [42,43,44,45,46], a rationale for engaging testosterone supplementation in rehabilitation practice should be provided. In addition, DHEA supplementation, which has been shown to increase levels of downstream sex steroids and, therefore, improves immune and stress response, glucose metabolism and body fat ratio may be an option to enhance the performance of existing rehabilitation strategies after the injury [47,48,49,50].

We observed significantly lower levels of DHEA and DHEA-S among opioid users. Previous studies reported a dose-related DHEA-S deficiency in adults who are chronically consuming sustained-action oral or transdermal opioids [51]. In our study, at baseline, >30% of study participants used opioids, while only 11% were discharged with those medications. Opioid-induced endocrinopathy is a common adverse effect of long-term opioid therapy [52]. In SCI individuals using opioids, symptoms of endocrinopathy (such as sexual dysfunction, decreased muscle mass, anemia, or low testosterone) may remain unrecognized and attributed to the injury. Thus, monitoring hormonal levels in this subpopulation may be crucial.

Our findings in women are only exploratory and were based on limited data. We observed lower levels of DHEA in women with non-traumatic as compared to traumatic injury. Furthermore, DHEA levels decreased during the rehabilitation period. In this study, we did not have information about menopausal or menstruation status and thus, this makes the interpretation of our findings challenging. Total testosterone levels were above 2.4 nmol/L among 37.5% of women at baseline. In women, hyperandrogenism was associated with an increase in the prevalence of several metabolic factors (dyslipidemia, insulin resistance, hypertension, obesity), which could lead to an increased risk of diabetes but also cardiovascular disease, and stroke [53]. Thus, a detailed hormonal status assessment in the context of both SCI and women-specific factors (such as menstruation, menopause, polycystic ovarian syndrome, etc.) is warranted to understand pathophysiological changes in metabolic milieu post-injury.

### 4.3. Strengths and Limitations of the Current Study

To our knowledge, this is the first study to explore longitudinal changes in androgen levels using linear mixed model approach, which is a robust and powerful tool for analyzing complex datasets with repeated or clustered observations. We adjusted our analyses for factors such as injury characteristics or medication use and we further restricted the analyses to individuals with traumatic injury and our results remain stable. In addition, we measured SHBG and other androgens such as DHEA and DHEA-S that were not often studied in the SCI population but were identified as key determinants of health and wellbeing in ABI [5,12,13,54,55,56].

Our study has some limitations that need to be mentioned. First, androgen levels were measured using ELISA rather than LC-MS, which is considered the gold diagnostic standard for steroid hormone monitoring. Although ELISA measurements have high correlation with LC-MS/MS within the adult male testosterone range, its precision in hypogonadal range is lower. Therefore, our measurements in this range may underestimate the true prevalence of low total and free testosterone. Our study, having a strictly research purpose did not aim to provide clinical diagnosis of hypogonadism. Instead, the main contribution of this work is the identification of changes in the longitudinal hormonal trajectories/during rehabilitation. Importantly, using ELISA, facilitates comparison of our findings to previous published works on the SCI population, where ELISA or radioimmunoassay (RIA) were the most commonly applied analytical methods. Furthermore, the ELISA kits from two manufacturers, Abnova and Abcam, were used to measure androgen levels, thus, the assessment of correlation between androgen levels was not feasible. Third, the SHBG assay sensitivity is not optimized for the natural range of the analyte and levels measured in current study cannot be compared to clinical standard (ELISA kit detection range was between 62.5 pg/mL and 4000 pg/mL). Finally, we were not able to explore the association between testicular blood flow, which was previously linked with testicular function and may be a crucial factor to influence androgen levels [57]. 

## 5. Conclusions

In this study we observed an improvement in total testosterone and DHEA-S in men over first inpatient rehabilitation. Androgens may play a pivotal role in functional recovery and early metabolic changes in SCI. Thus, to develop timely preventive strategies, future methodologically sound longitudinal studies are required to disentangle the complex association between hormone levels and aging, visceral adiposity, physical inactivity and functional recovery post-injury. 

## Figures and Tables

**Table 1:** Baseline Characteristics of the SCI individuals (beginning of rehabilitation).

Characteristic	All Observations (n = 86)	Males (n = 70, 81 %)	Females (n = 16, 19 %)	*p* Value ^1^
Age, years, median (IQR)	51 (36–64)	52.5 (35–63)	47 (38–73.5)	0.68
Injury characteristics				
Tetraplegia, n (%)	29 (33.7)	24 (34.3)	5 (31.3)	0.19
Paraplegia, n (%)	43 (50)	37 (52.9)	6 (37.5)	
Missing, n (%)	14 (16.3)	9 (12.9)	5 (31.3)	
Time since injury, days	15.5 (10–27)	16 (10–27)	12.5 (8.5–24.5)	0.48
Length of rehabilitation, months	5.6 (4.2–7.5)	5.7 (4.2–7.5)	5.1 (4.3–5.9)	0.29
Cause of injury				
Traumatic, n (%)	61 (70.9)	52 (74.3)	9 (56.2)	0.15
Non-traumatic, n (%)	25 (29.1)	18 (25.7)	7 (43.8)	
Completeness of injury				
Motor Complete	33 (38.4)	29 (41.4)	4 (25.0)	0.16
Motor Incomplete	39 (45.4)	32 (45.7)	7 (43.7)	
Missing	14 (16.3)	9 (12.9)	5 (31.3)	
Cause of traumatic injury				
Vehicular	22 (36.1)	22 (42.3)	0 (0)	0.03
Violence	1 (1.6)	0 (0)	1 (11.1)	
Sports	17 (27.9)	12 (23.1)	5 (55.6)	
Falls	15 (24.6)	13 (25.0)	2 (22.2)	
Medical complication	5 (8.2)	4 (7.7)	1 (11.1)	
Unknown	1 (1.6)	1 (1.9)	0 (0)	
Cause of non-traumatic injury				
Genetic disorders	8 (32.0)	5 (27.8)	3 (42.9)	0.80
Metabolic	9 (36.0)	6 (33.3)	3 (42.9)	
Toxic	6 (24.0)	5 (27.8)	1 (14.3)	
Infections	1 (4.0)	1 (5.6)	0	
Miscellaneous	1 (4.0)	1 (5.6)	0	
ISNCSCI Scale				
A, n (%)	19 (26.4)	17 (27.9)	2 (18.2)	0.69
B, n (%)	14 (19.4)	12 (19.7)	2 (18.2)	
C, n (%)	11 (15.3)	10 (16.4)	1 (9.1)	
D, n (%)	28 (38.9)	22 (36.1)	6 (54.6)	
Anthropometric measurements				
BMI, kg/m^2^	23.7 (21.3–26.3)	23.7 (21.5–26.0)	23.4 (20.4–26.3)	0.76
Waist circumference, cm	86.5 (79.3–96)	87 (79.5–97)	83 (78–90)	0.63
Androgens				
Total testosterone, nmol/L, median (IQR)	10.4 (5.3–17.3)	12.5 (7.9–17.7)	1.9 (1.4–2.5)	<0.01
Free testosterone, pmol/L, median (IQR)	24.1 (11.5–33.0)	27.5 (16.9–36.6)	2.9 (2.3–3.7)	<0.01
SHBG, pg/mL, median (IQR) ^3^	2606 (2053–2944)	2558 (2053–2777)	2890 (2020.5–3947)	0.11
DHEA, nmol/L, median (IQR)	19.7 (13.5–38.0)	20.2 (12.8–40.2)	18.9 (15.7–35.0)	0.50
DHEA-S, umol/L, median (IQR)	3.3 (1.6–6.5)	3.6 (1.6–6.9)	3.1 (1.5–4.6)	0.35
Medication use				
Opioids, n (%)	26 (30.2)	23 (32.9)	3 (18.8)	0.27
Corticosteroids, n (%)	8 (9.3)	7 (10.0)	1 (6.3)	0.64
Thyroid hormones, n (%)	4 (4.7)	2 (2.9)	2 (12.5)	0.10
Comorbidities				
Cardiovascular diseases ^2^, n (%)	4 (4.7)	2 (2.9)	2 (12.5)	0.10
Type 2 diabetes/prediabetes, n (%)	6 (7.0)	4 (5.7)	2 (12.5)	0.34
Hypertension, n (%)	8 (9.3)	7 (10.0)	1 (6.3)	0.64
Chronic kidney disease, n (%)	3 (3.5)	1 (1.4)	2 (12.5)	0.03

Normal ranges according to manufacturer: Total Testosterone-Males (6.94–23.94 nmol/L), females (0.90–4.23 nmol/L); Free testosterone-Men (15.6–145.7 pmol/L), Women (0.35–14.2 pmol/L); SHBG: NA, DHEA: NA, DHEA-S-Men (2.71–11.40 umol/L), Women (0.27–10.58 umol/L). ^1^ For difference between men and women using the Student’s *t*-test, Wilcoxon signed rank test and chi-square test, as appropriate. ^2^ Cardiovascular diseases are defined are those with chronic stable angina, acute coronary syndrome (N/STEMI and unstable angina), venous thrombosis (deep vein thrombosis and pulmonary embolism), post-operative procedure for cardiovascular diseases (stents or bypass), and other non-coronary atherosclerotic diseases (i.e., aortic aneurysm, peripheral artery disease, carotid artery disease). ^3^ the ELISA kit used for research purposes targets the mature, full-length, protein form which lacks the N-terminal signaling peptide. Abbreviations: BMI: body mass index, DHEA: Dehydroepiandrosterone, DHEA-S: Dehydroepiandrosterone sulfate, ISNCSCI: International Standards for Neurological Classification of SCI, SHBG: Sex Hormone Binding Globulin.

**Table 2:** Association between individual patient and injury’s characteristics and hormone levels at baseline (beginning of rehabilitation).

Men (N = 70)					
Parameter	TT (β, 95%CI)	FT (β, 95%CI)	SHBG (β, 95%CI)	DHEA (β, 95%CI)	DHEA-S (β, 95%CI)
Etiology of injury					
Non-traumatic	2.30 (−2.02, 6.61)	21.46 (−67.1, 110.00)	−167.28 (−544.62, 210.06)	6.78 (−4.4, 18.00)	−0.09 (−1.97, 1.80)
Injury level					
Paraplegics	2.07 (−1.67, 5.79)	15.30 (−64.58, 95.17)	88.56 (−291.17, 468.29)	−2.32 (−11.39, 6.76)	−1.77 (−3.60, 0.05)
Injury completeness					
Incomplete	2.25 (−1.61, 6.12)	21.32 (−55.33, 97.98)	179.33 (−200.05, 558.70)	−3.18 (−11.97, 5.61)	−1.78 (−3.43, −0.12) *
**Medication use**					
Opioids use	1.97 (−1.87, 5.80)	−56.40 (−126.62, 13.82)	557.76 (281.48, 834.03) ***	−9.02 (−17.01, −1.03) *	−2.18 (−3.58, −0.78) **
Corticosteroids use	4.51 (0.08, 8.94)	45.99 ( −52.07, 144.05)	31.60 (−568.62, 631.82)	3.66 (−12.15, 19.47)	0.02 (−1.95, 2.00)
**Women (N = 16)**					
**Parameter**	**TT** **(β, 95%CI)**	**FT** **(β, 95%CI)**	**SHBG** **(β, 95%CI)**	**DHEA** **(β, 95%CI)**	**DHEA-S** **(β, 95%CI)**
Etiology of injury					
Non-traumatic	0.36 (−1.91, 2.63)	4.65 (−21.98, 31.29)	55.14 (−1131.25, 1241.54)	−18.05 (−35.51, −0.58) *	−1.32 (−3.91, 1.27)
Injury level					
Paraplegics	0.97 (−1.98, 3.92)	10.47 (−21.48, 42.42)	132.93 (−1428.11, 1693.97)	0.32 (−18.78, 19.42)	−1.61 (−4.05, 0.83)
Injury completeness					
Incomplete	0.12 (−2.54, 2.77)	9.02 (−18.74, 36.77)	−416.18 (−1815.78, 983.43)	−12.79 (−29.09, 3.51)	−0.19 (−2.49, 2.12)
Medication use					
Opioids use	0.21 (−1.29, 1.71)	−4.54 (−20.29, 11.22)	1158.41 (310.59, 2006.23) *	−5.70 (−22.46, 11.07)	−1.93 (−3.95, 0.08)
Corticosteroids use	0.38 (−0.71, 1.47)	−5.96 (−18.93, 7.00)	388.13 (−248.53, 1024.80)	7.31 (−3.97, 18.59)	−0.24 (−1.70, 1.22)

TT: Total testosterone, FT: Free testosterone, SHBG: Sex Hormone Binding Globulin, DHEA: Dehydroepiandrosterone, DHEA-S: Dehydroepiandrosterone sulfate, β = Beta estimate from linear regression. (Traumatic SCI, Tetraplegics and Complete SCI served as reference groups). * *p* value < 0.05 ** *p* < 0.01 *** *p* < 0.001.

**Table 3:** Longitudinal changes in androgens.

	Admission to Rehabilitation	End of Rehabilitation	*p*-Value	Unadjusted Model	*p*-Value	Adjusted Model ^1^	*p*-Value
	Mean (SD)	Mean (SD)	(Paired *t* Test)	(β, 95%CI)	(LMM)	(β, 95%CI)	(LMM)
Men (N = 70)							
TT, nmol/L	13.4 (7.2)	17.0 (6.2)	<0.01	3.66 (1.87, 5.46)	<0.01	3.96 (1.37, 6.56)	0.003
FT, pmol/L	29.4 (15.8)	30.8 (18.5)	0.32	13.85 (−26.89, 54.59)	0.51	2.99 (−53.56, 59.53)	0.92
SHBG, pg/mL	2360.12 (744.88)	2294.10 (706.31)	0.12	−66.01 (−140.40, 8.37)	0.08	−37.61 (−145.04, 69.81)	0.49
DHEA, nmol/L	26.2 (17.6)	26.9 (21.9)	0.60	0.65 (−3.20, 4.49)	0.74	−1.59 (−7.39, 4.21)	0.59
DHEA-S, umol/L	4.5 (3.3)	5.9 (4.4)	<0.01	1.44 (0.76, 2.12)	<0.01	1.77 (0.73, 2.81)	<0.01
**Women (N = 16)**							
TT, nmol/L	2.1 (1.7)	2.2 (1.9)	0.80	0.06 (−0.47, 0.60)	0.82	0.23 (−0.34, 0.79)	0.43
FT, pmol/L	3.4 (2.2)	3.1(3.2)	0.54	−2.09 (−11.78, 7.60)	0.67	2.71 (−10.22, 15.65)	0.68
SHBG, pg/mL	2770.13 (1079.79)	2767.06 (903.71)	0.60	−3.06 (−322.10, 315.97)	0.99	514.08 (−87.06, 1115.23)	0.09
DHEA, nmol/L	28.2 (18.3)	21.1 (14.0)	0.05	−7.11 (−13.98, −0.24)	0.04	−11.91 (−24.39, −4.11)	<0.01
DHEA-S, umol/L	3.4 (2.5)	4.4 (3.6)	0.29	1.01 (−0.31, 2.32)	0.13	2.71 (−0.02, 5.43)	0.05

SHBG: Sex Hormone Binding Globulin, DHEA: Dehydroepiandrosterone, DHEA-S: Dehydroepiandrosterone sulfate, B = Beta estimate from linear mixed model (LMM), 95%CI (95% Confidence Interval). ^1^ Adjusted for age, BMI, level and completeness of injury, time since injury, opioid and corticosteroid use.

## Data Availability

The data sets generated and/or analyzed during the current study are available from the corresponding author on reasonable request.

## Supplementary Materials

The following supporting information can be downloaded at: https://www.mdpi.com/article/10.3390/jcm11216559/s1, Figure S1. Sexual dysfunction as reported by study participants at T2 (*70–98 days post-injury*) Figure S2: Association between age and androgens in men; Figure S3: Association between age and androgens in women; Figure S4: Association between age and androgens in men with motor complete traumatic SCI; Figure S5: Association between time since injury and androgens in men; Figure S6: Association between time since injury and androgens in women; Figure S7: Bivariate plots of changes in androgen sex hormone levels in men with spinal cord injury; Figure S8: Bivariate plots of changes in androgen sex hormone levels in women with spinal cord injury; Table S1: Detailed description of hormone measurements; Table S2: Causes of Non-traumatic spinal cord injury; Table S3: Association between individual patient and injury’s characteristics and hormone levels at baseline, analysis restricted to men with traumatic injury; Table S4: Longitudinal changes in androgens, analysis restricted to men with traumatic spinal cord injury; Table S5: Longitudinal changes in sex hormones (Wilcoxon sign test and Linear mixed models).
