# Supplementary material for: Early Changes in Androgen Levels in Individuals with Spinal Cord Injury: A Longitudinal SwiSCI Study
Source: J Clin Med. 2022 Nov 4;11(21):6559. doi: 10.3390/jcm11216559 (PMC9655492; doi:10.3390/jcm11216559)
Supplement: Supplementary file 1 [file jcm-11-06559-s001.zip › jcm-1982377-supplementary.pdf]

1. ONLINE SUPPLEMENTARY

Content

1. ONLINE SUPPLEMENT 1
Table S1. Detailed description of hormone measurements 2
Table S2. Causes of Non-traumatic spinal cord injury 4
Table S3. Association between individual patient and injury's characteristics and hormone levels at baseline, analysis restricted to men with traumatic injury 5
Table S4. Longitudinal changes in androgens, analysis restricted to men with traumatic spinal cord injury 6
Table S5. Longitudinal changes in sex hormones (Wilcoxon sign test and Linear mixed models) 7
Figure S1. Sexual dysfunction as reported by study participants at T2 (70–98 days post-injury) 8
Figure S2. Association between age and androgens in men with spinal cord injury 9
Figure S3. Association between age and androgens in women with spinal cord injury 10
Figure S4. Association between age and androgens in men with motor complete traumatic SCI 11
Figure S5. Association between time since injury and androgens in men with spinal cord injury 12
Figure S6. Association between time since injury and androgens in women with spinal cord injury 13
Figure S7. Bivariate plots of changes in androgen sex hormone levels in men with spinal cord injury 14
Figure S8. Bivariate plots of changes in androgen sex hormone levels in women with spinal cord injury 15

Table S1. Detailed description of hormone measurements								
**Sex Hormone**	**Assay[1]**	**Manufacturer/supplier**	**Type of standard curve**	**Volume of sample used**	**Lower detection limit**	**Intra-Assay Coefficient of variability (CV)**	**Inter-assay coefficient of variability (CV)**	**Normal range according to manufacturer**
Total testosterone (TT)	Competitive Enzyme Linked Immunosorbent Assay (ELISA)	Abcam, Lucerna-Chem AG, Switzerland (cat.no: ab174569)	7-point standard curve in duplicate	25 µl of serum per well for 40 samples in duplicate (80 wells)	0.0083 ng/ml (0.0288 nmol/L)	4.16%, 3.28%, 3.34% for 0.73ng/ml (2.53 nmol/L), 4.88ng/ml (16.92 nmol/L), and 11.26ng/ml (39.04 nmol/L) mean values of TT respectively	<10%	6.94-23-94 nmol/L for males and 0.90-4.23 nmol/L for females.
Free testosterone (FT)	Competitive Enzyme Linked Immunosorbent Assay (ELISA)	Abcam, Lucerna-Chem AG, Switzerland (cat.no:ab178663)	6-point standard curve including positive and negative controls in duplicate	20 µl of serum per well for 38 samples in duplicate (76 wells)	0.06 pg/ml (0.21 pmol/L)	<10%	<10%	4.5-42 pg/ml (15.6-145.7 pmol/L) for males and 0.1-4.1 pg/ml (0.35-14.2 pmol/L) for females
Sex Hormone Binding Globulin (SHBG)	Sandwich Enzyme Linked Immunosorbent Assay (ELISA)	Abcam, Lucerna-Chem AG, Switzerland (SHBG, cat.no:ab260070)	7-point standard curve in duplicate	50 µl of serum per well for 40 samples in duplicate (80 wells)	26 pg/ml (0.023 nmol/L)	3.8%	4.6%	No recommended physiological reference ranges were provided by the kit manufacturer
Dehydroepiandosterone (DHEA)	Competitive Enzyme Linked Immunosorbent Assay (ELISA)	Abnova, Lucerna-Chem AG, Switzerland (cat.no:KA0315)	7-point standard curve including non-specific binding, zero activity, and total activity wells in duplicate	100 µl of serum per well for 36 samples in duplicate (72 wells)	2.9 pg/ml (0.01 nmol/L)	<10%	<10%	No recommended physiological reference ranges were provided by the kit manufacturer

Dehydroepiandosterone sulphate (DHEAs)	Competitive Enzyme Linked Immunosorbent Assay (ELISA)	Abnova, Lucerna-Chem AG, Switzerland (DHEA-S, cat.no:KA0920)	7-point standard curve was run in duplicate	10 µl of serum per well for 40 samples in duplicate.	0.0236 ug/ml (0.06 umol/L)	<5%	<5%	normal ranges in males and females are 1.0-4.2 µg/mL (2.71-11.40 umol/L) and 0.1 to 3.9 ug/ml (0.27-10.58 umol/L) respectively according to manufacturer`s guidelines

[1] The assays were performed on 96 well plates.

Table S2. Causes of Non-traumatic spinal cord injury

**Medical condition**	**Frequency n= 25 (%)**
Spinal cord compression due to combination of multiple factors	3 (12)
Neoplasm	3 (12)
Bacterial infection	1 (4)
Spinal stenosis	4 (16)
Demyelination	1 (4)
Malignant neoplasm	1 (4)
Vascular malformations	2 (8)
Vertebral column degenerative disorders	1 (4)
Viral infection	1 (4)
Inflammatory and Auto-immune Diseases	1 (4)
Vascular disorders (unknown)	1 (4)
Vascular disorders (Ischaemia)	3 (12)
Vascular disorders (Haemorrhage)	1 (4)
Infection (unknown)	1 (4)
Miscellaneous (Unknown)	1 (4)

Table S3. Association between individual patient and injury's characteristics and hormone levels at baseline, analysis restricted to men with traumatic injury

	Men with traumatic injury (N=52)				
	**Total testosterone (β, 95% CI)**	**Free Testosterone (β, 95% CI)**	**SHBG (β, 95% CI)**	**DHEA (β, 95% CI)**	**DHEAs (β, 95% CI)**
**Injury level**					
Paraplegics	1.58 (-2.66, 5.81)	31.29 (-52.16, 114.75)	25.25 (-426.65, 477.15)	-3.01 (-12.97, 6.96)	-1.53 (-3.61,0.55)
**Injury completeness**					
incomplete	3.38 (-0.85, 7.62)	36.64 (-54.83, 128.11)	220.35 (-248.93, 689.63)	**-10.05 (-19.63, -0.46)***	**-2.04 (-3.93, -0.15**)*
**Medication use**					
Opioids use	3.03 (-1.18, 7.24)	-49.90 (-132.50, 32.70)	**559.52(208.09, 910.94) ** **	-8.09 (-16.35, 0.16)	**-2.57 (-4.16, -0.99)****
Corticosteroids use	3.38 (-1.83, 8.59)	85.30 (-42.95, 213.54)	102.09 (-611.27, 815.46)	17.47 (-8.44, 43.38)	2.05 (-0.84, 4.94)

*P value<0.05 ** P<0.01 *** P<0.001
¥analysis was not possible due to small number of corticosteroid users among individuals with traumatic motor complete injury
Abbreviations: TT: Total testosterone, SHBG: Sex Hormone Binding Globulin, DHEA: Dehydroepiandrosterone, DHEAs: Dehydroepiandrosterone sulfate, B=Beta estimate from linear regression
(Tetraplegics and complete SCI served as reference)

Table S4. Longitudinal changes in androgens, analysis restricted to men with traumatic spinal cord injury

	**Beginning of Rehabilitation mean (SD)**	**End of Rehabilitation mean (SD)**	**p-value (paired t test)**	**Unadjusted model (β, 95% CI)**	**p-value (LMM)**	**Model 1 (β, 95% CI)**	**p-value (LMM)**
**Men (N= 52)**							
Total testosterone, nmol/L	12.8 (6.8)	16.8 (5.5)	**0.0007**	4.04 (1.85, 6.23)	**<0.0001**	4.69 (1.53, 7.84)	**0.004**
Free testosterone, pmol/L	288.1 (156.9)	302.3 (185.6)	0.56	14.23 (-32.97, 61.42)	0.56	24.07 (-42.15, 90.29)	0.48
SHBG, nmol/L	2.1 (0.7)	2.1 (0.6)	0.10	-0.06 (-0.14, 0.01)	0.10	-0.005 (-0.11, 0.10)	0.92
DHEA, nmol/L	24.5 (15.5)	24.5 (19.7)	1.00	0.003 (-4.54, 4.55)	1.00	-1.05 (-8.21, 6.11)	0.77
DHEAS, umol/L	4.5 (3.3)	5.9 (4.4)	**0.002**	1.43 (0.57, 2.29)	**0.001**	1.93 (0.59, 3.26)	**0.005**
Model 1: Adjusted for age, BMI, injury level, completeness of the injury, time since injury, opioids and corticosteroids use							

Table S5. Longitudinal changes in sex hormones (Wilcoxon sign test and Linear mixed models)

	Beginning of Rehabilitation Median (IQR)	End of Rehabilitation Median (IQR)	p-value (Wilcoxon Sign test)	Unadjusted model¨ (β, 95% CI;)	p-value (LMM)	Model 1 (β, 95% CI;)	p-value (LMM)
**Men (N= 70)**							
Total testosterone, nmol/L	12.5(7.9-17.7)	16.4(11.7-19.8)	**0.02**	3.66(1.87,5.46)	<0.0001	3.66(1.87,5.46)	**<0.0001**
Free testosterone, pmol/mL	274.7(169.3-366.4)	268.0(187.6-362.69	0.55	13.85(-26.89, 54.59)	0.51	13.85(-26.89, 54.59)	0.51
SHBG, pg/ml	2558 (2053-2777)	2470 (2056-2829)	1.00	-66.01(-140.40, 8.37)	0.08	-66.01(-140.40, 8.37)	0.08
DHEA, nmol/L	20.2(12.8- 40.2)	19.9(12.6-36.6)	0.91	0.65(-3.20,4.49)	0.74	0.65(-3.20,4.49)	0.74
DHEAS, umol/L	3.6(1.6-6.9)	4.7(2.2-8.2)	**0.001**	1.44(0.76, 2.12)	<0.0001	1.44(0.76, 2.12)	**<0.0001**
**Women (N=16)**							
Total testosterone, nmol/L	1.9(1.4-2.5)	1.7(1.3-2.6)	1.00	0.06(-0.47,0.60)	0.82	0.07 (-0.47,0.61)	0.80
Free testosterone, pmol/mL	29.4(23.3-37.3)	25.5(12.3-36.9)	1.00	-2.09(-11.78, 7.60)	0.67	-2.09(-11.78, 7.60)	0.67
SHBG, pg/ml	2890 (2020.5-3947)	2916 (1936-3445.5)	1.00	-3.06 (-322.10, 315.97)	0.99	-3.06 (-322.10, 315.97)	0.99
DHEA, nmol/L	18.9(15.7-35.0)	17.9(12.3-32.2)	0.61	-7.11(-13.98,-0.24)	**0.04**	-7.11(-13.98,-0.24)	**0.04**
DHEAS, umol/L	3.1(1.5-4.6)	2.7(1.4-7.2)	0.80	1.01(-0.31, 2.32)	0.13	1.01(-0.31, 2.32)	0.13

Model 1: Adjusted for age

Figure S1. Sexual dysfunction as reported by study participants at T2 (70–98 days post-injury)

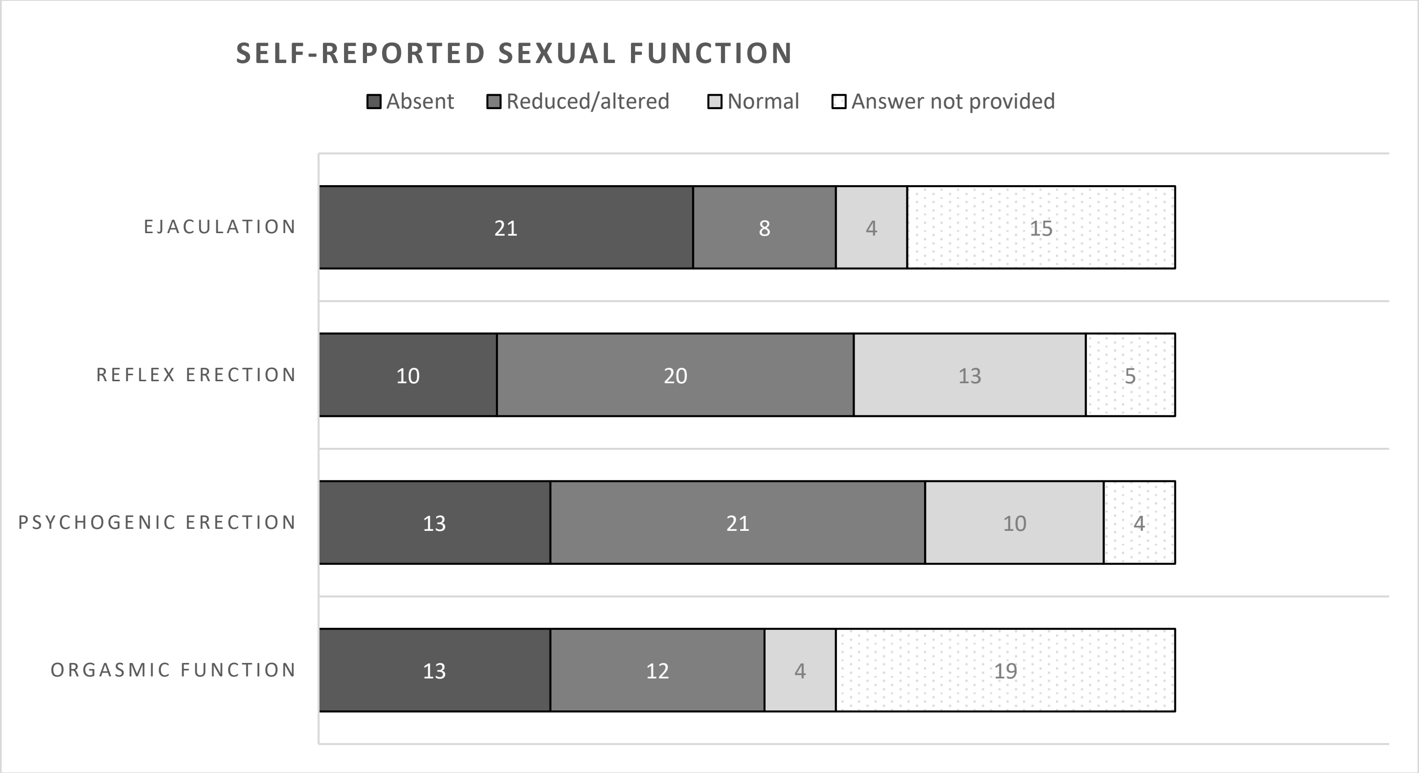

Figure S2. Association between age and androgens in men with spinal cord injury

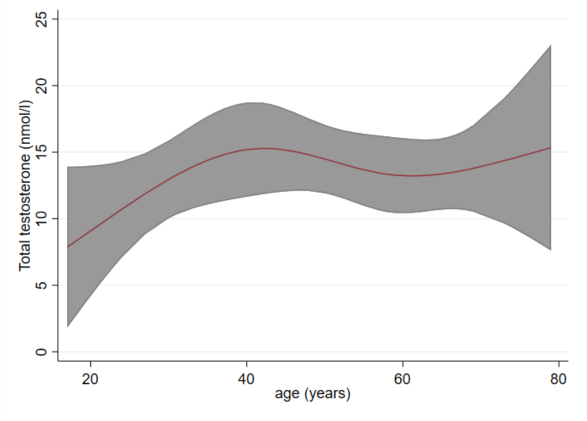

A. Total testosterone

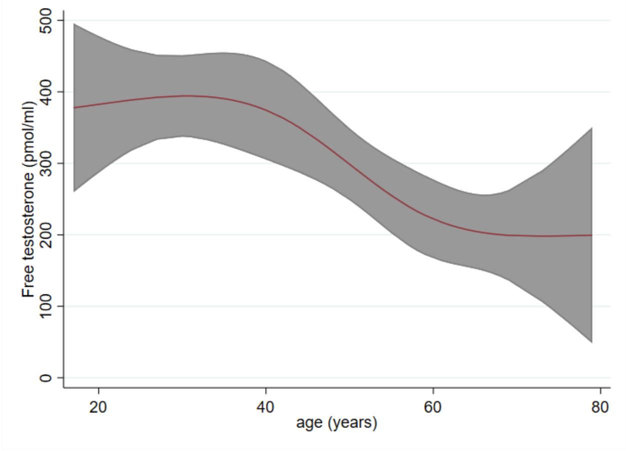

B. Free testosterone

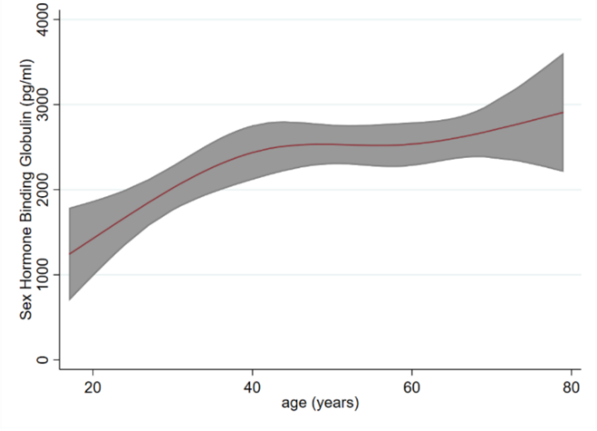

C. Sex Hormone Binding Globulin

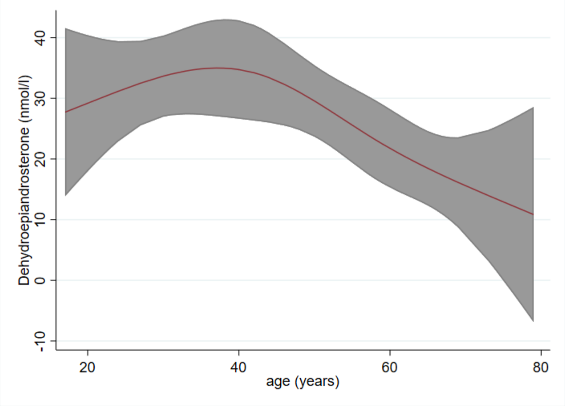

D. Dehydroepiandrosterone

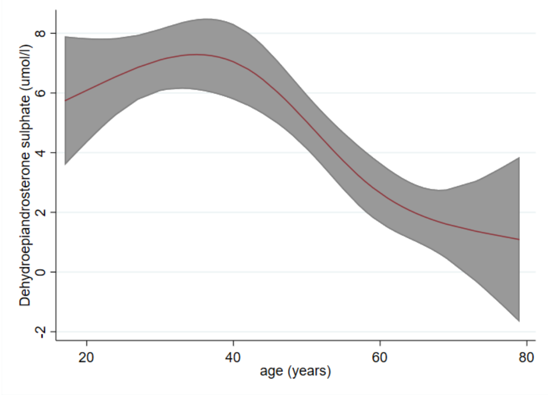

E. Dehydroepiandrosterone sulphate

Figure S3. Association between age and androgens in women with spinal cord injury

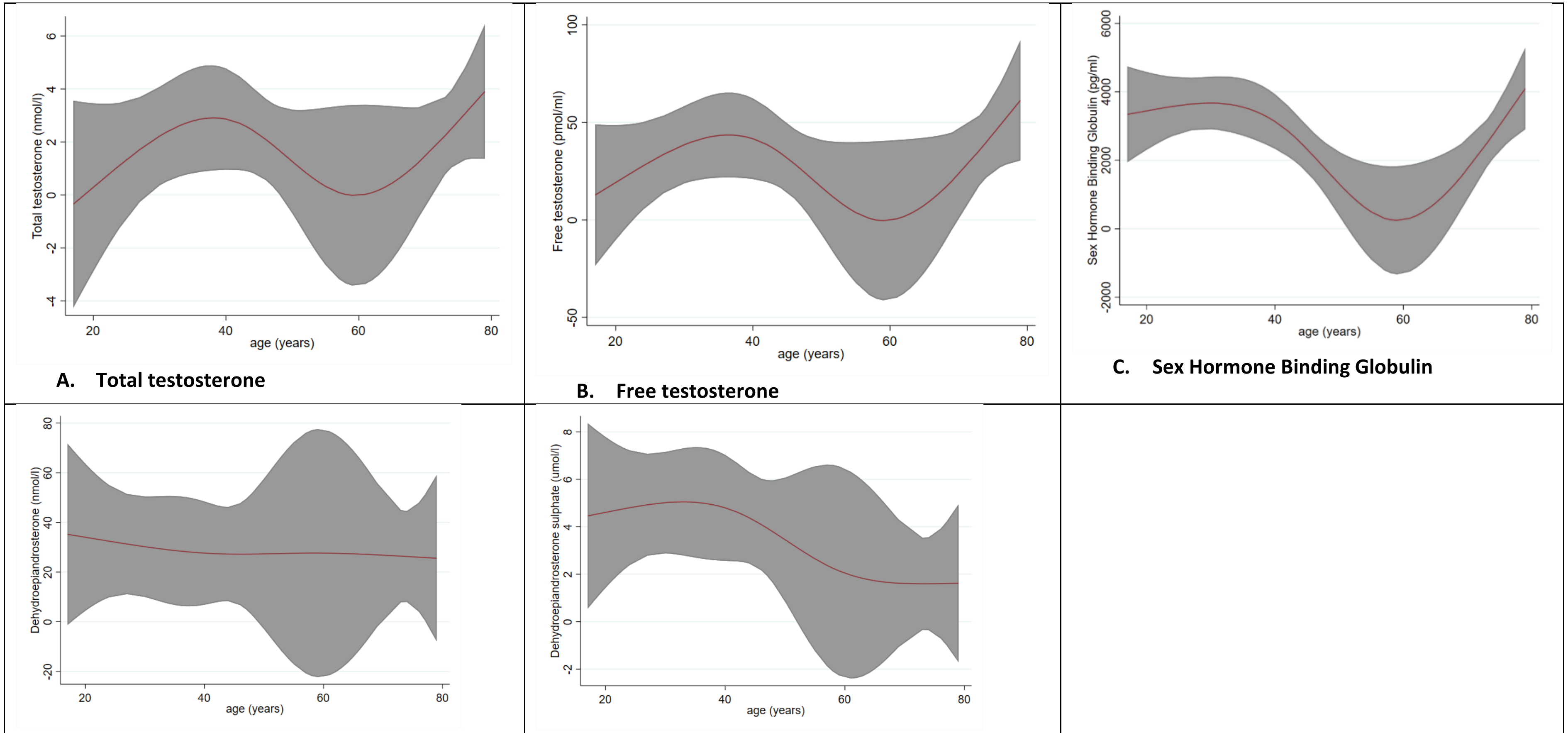

A. Total testosterone

B. Free testosterone

C. Sex Hormone Binding Globulin

D. Dehydroepiandrosterone

E. Dehydroepiandrosterone sulphate

Figure S4. Association between age and androgens in men with motor complete traumatic SCI

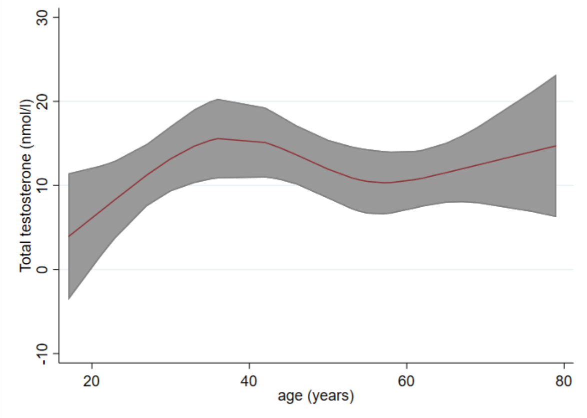

A. Total testosterone

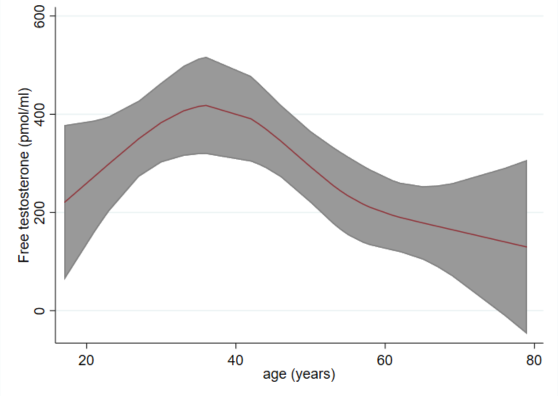

B. Free testosterone

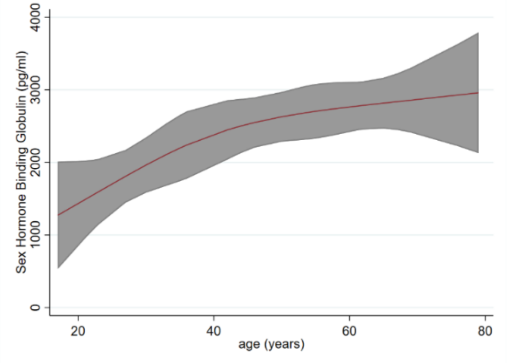

C. Sex Hormone Binding Globulin

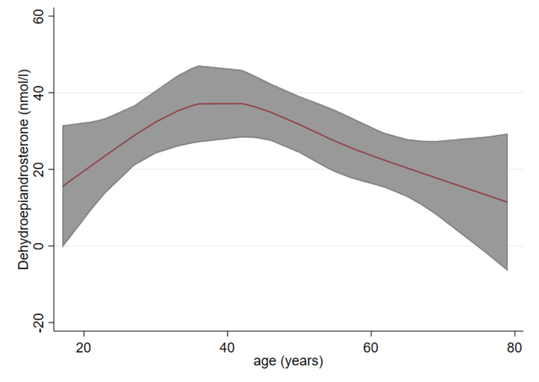

D. Dehydroepiandrosterone

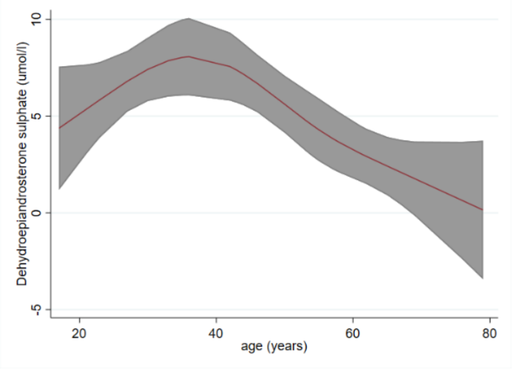

E. Dehydroepiandrosterone sulphate

Figure S5. Association between time since injury and androgens in men with spinal cord injury

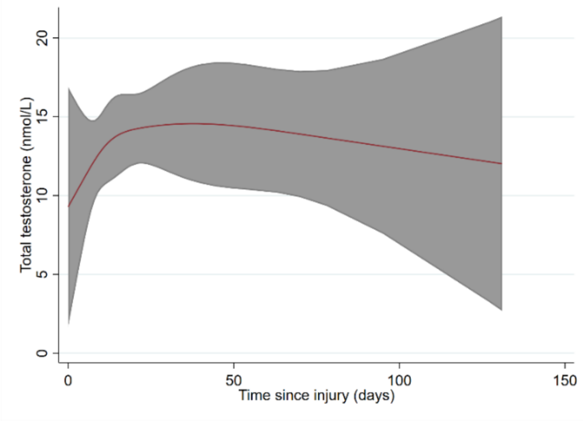

A. Total testosterone

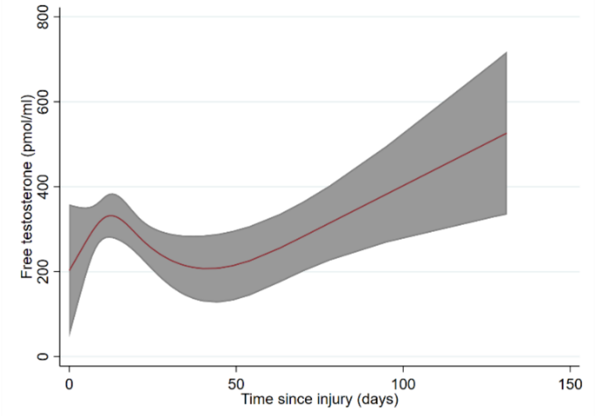

B. Free testosterone

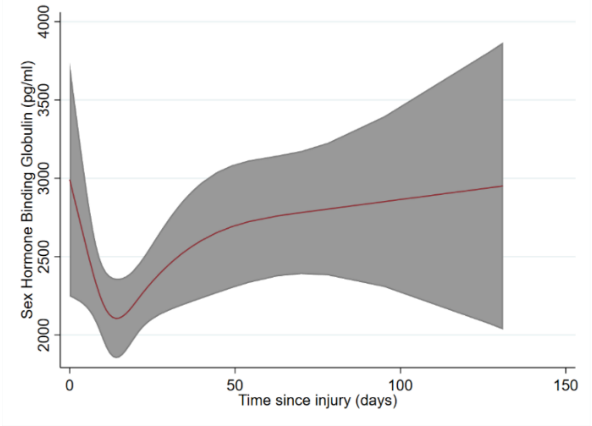

C. Sex hormone binding globulin

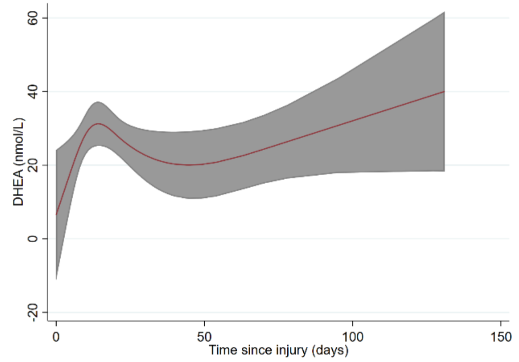

D. Dehydroepiandrosterone

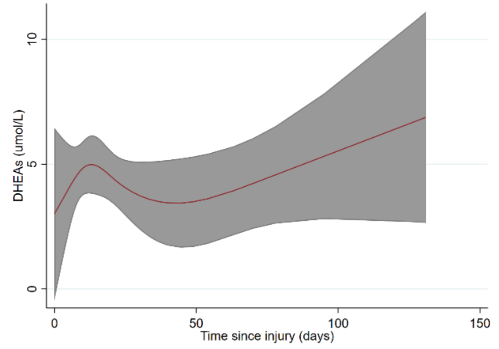

E. Deydroepiandrosterone sulphate

Figure S6. Association between time since injury and androgens in women with spinal cord injury

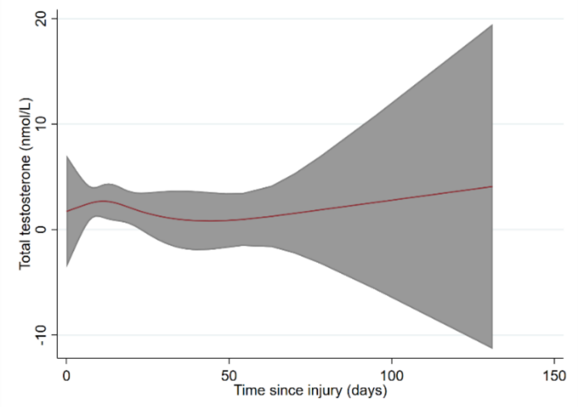

A. Total testosterone

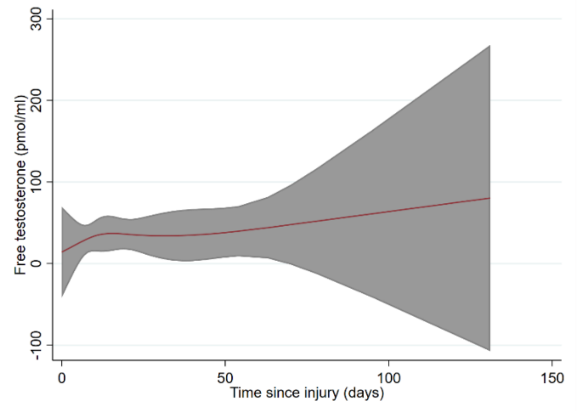

B. Free testosterone

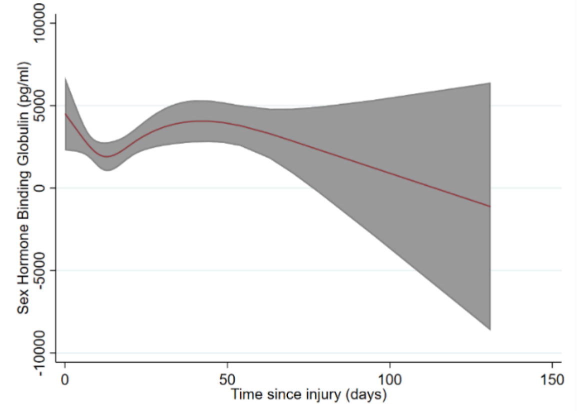

C. Sex hormone binding globulin

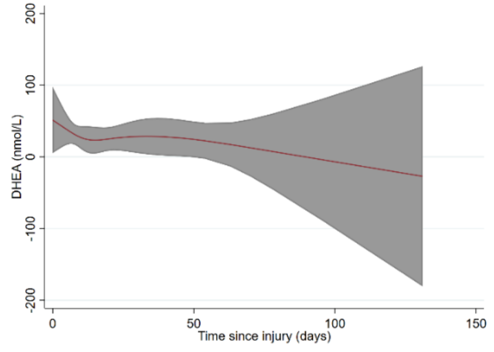

D. Dehydroepiandrosterone

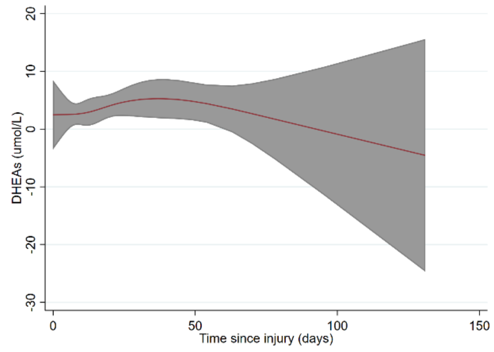

E. Deydroepiandrosterone sulphate

Figure S7. Bivariate plots of changes in androgen sex hormone levels in men with spinal cord injury

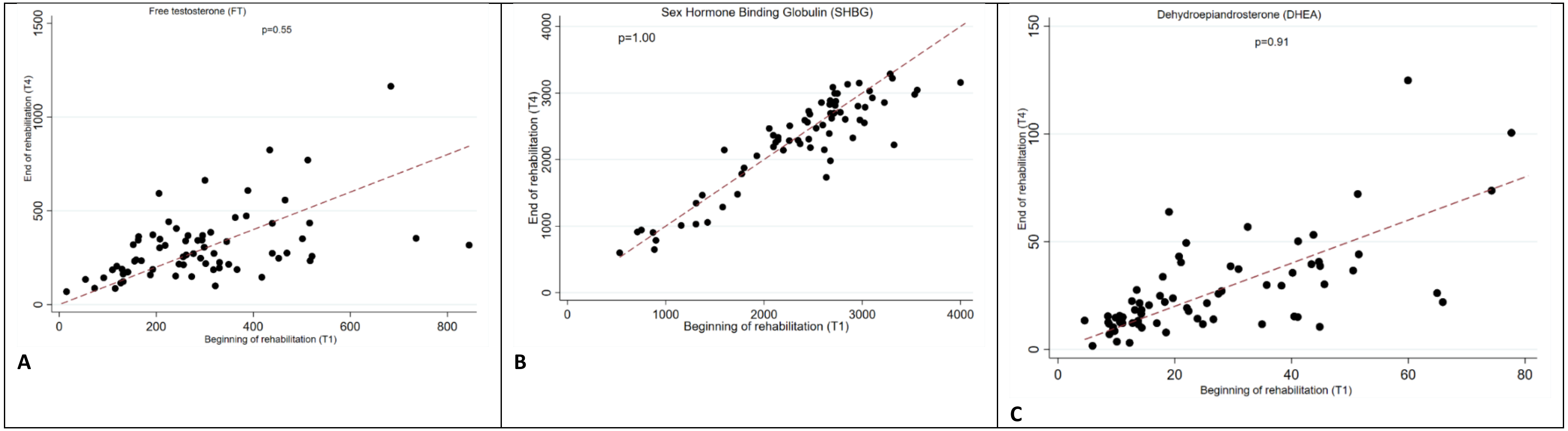

The bivariate plot shows individual changes in total testosterone and DHEA-S comparing the beginning and end of the rehabilitation period. A dotted line represents a line of no change. Every circle represents a person. A circle above the line indicates that a person had a higher level of androgen at follow-up as compared to baseline, whereas a circle below the line indicates that a person had a lower level of androgen at follow-up as compared to baseline.

Figure S8. Bivariate plots of changes in androgen sex hormone levels in women with spinal cord injury

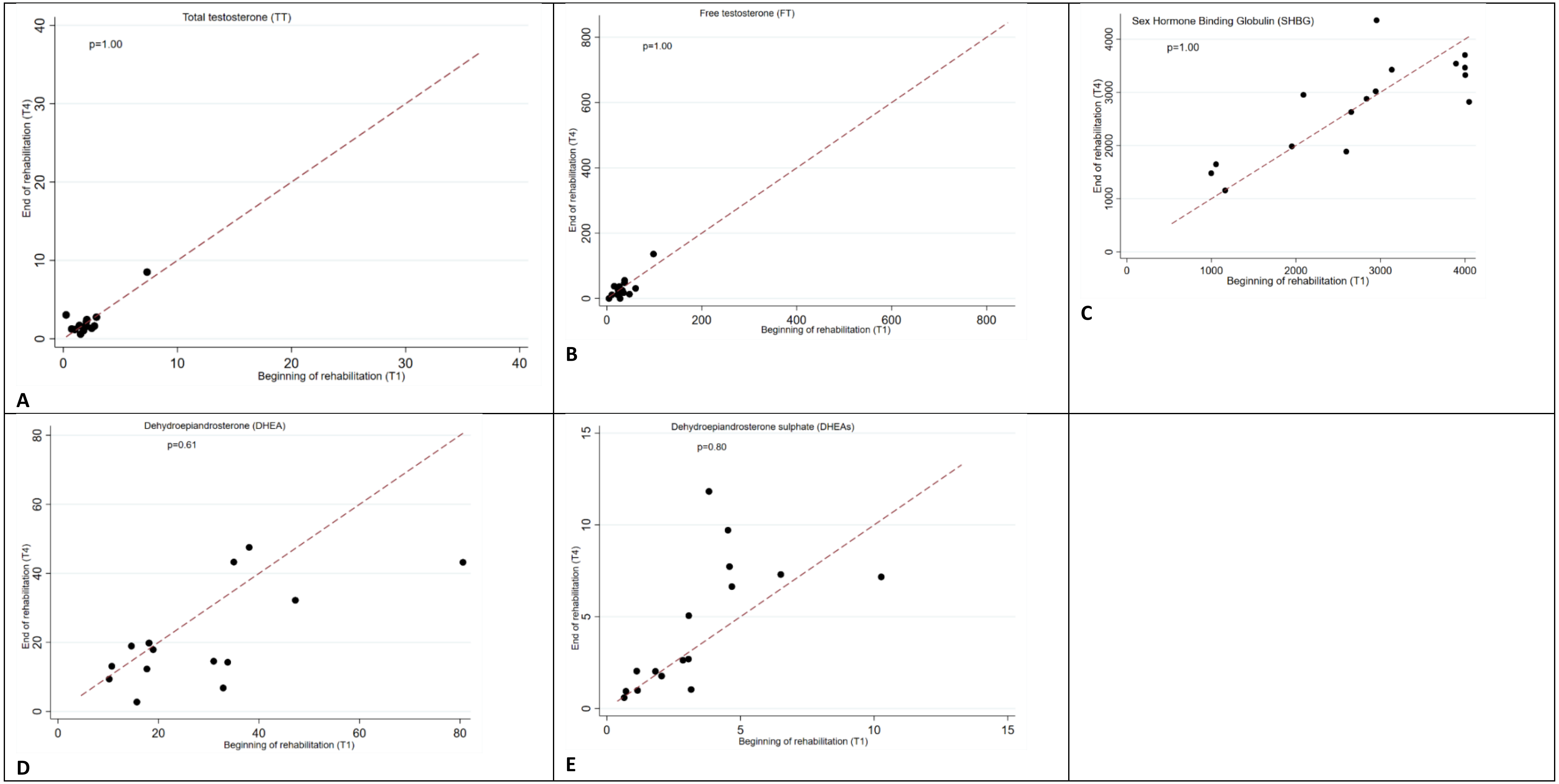

The bivariate plot shows individual changes in total testosterone and DHEA-S comparing the beginning and end of the rehabilitation period. A dotted line represents a line of no change. Every circle represents a person. A circle above the line indicates that a person had a higher level of androgen at follow-up as compared to baseline, whereas a circle below the line indicates that a person had a lower level of androgen at follow-up as compared to baseline.
